# Adult Primary Bone Sarcoma and Time to Treatment Initiation: An Analysis of the National Cancer Database

**DOI:** 10.1155/2018/1728302

**Published:** 2018-11-11

**Authors:** Joshua M. Lawrenz, Gannon L. Curtis, Joseph F. Styron, Jaiben George, Peter M. Anderson, Stacey Zahler, Dale R. Shepard, Brian P. Rubin, Lukas M. Nystrom, Nathan W. Mesko

**Affiliations:** ^1^Orthopaedic and Rheumatologic Institute, Cleveland Clinic, Cleveland, OH 44195, USA; ^2^Taussig Cancer Institute, Cleveland Clinic, Cleveland, OH 44195, USA; ^3^Robert J. Tomsich Pathology and Laboratory Medicine Institute, Cleveland Clinic, Cleveland, OH 44195, USA

## Abstract

**Objective:**

The time to treatment interval (TTI), defined as the period from diagnosis to first definitive treatment, has very limited descriptions toward understanding delays in primary bone sarcoma (PBS) care. Our primary goal was to determine the national standard for time to treatment initiation (TTI) in PBS in adults and to identify characteristics associated with TTI variability.

**Methods:**

An analysis of the National Cancer Database identified 15,083 adult patients with PBS diagnosed from 2004 to 2013. Kruskal–Wallis analysis identified differences between covariates regarding TTI and regression modeling identified covariates that independently influenced TTI.

**Results:**

The median TTI was 22 days. Approximately 60% of patients were definitively treated in the same center where the index diagnosis was made. Increased TTI was correlated with a transition in care institution (incidence rate ratio (IRR) = 1.89; *P* < 0.001), being uninsured (IRR = 1.36; *P* < 0.001), primary tumor site in the pelvis (IRR = 1.26; *P* < 0.001), Medicaid insurance status (IRR = 1.22; *P* < 0.001), care at an academic center (IRR = 1.14; *P* < 0.001), non-white race (IRR = 1.12; *P*=0.002), and Medicare insurance status (IRR = 1.08; *P*=0.017). Decreased TTI was correlated with a diagnosis of chondrosarcoma (IRR = 0.85; *P* < 0.001), having surgery as the index treatment (IRR = 0.88; *P* < 0.001), a primary tumor site of the lower (IRR = 0.91; *P*=0.001) or upper extremity (IRR = 0.92; *P*=0.023), and stage II or stage III disease (IRR = 0.91; *P*=0.010).

**Conclusions:**

TTI is associated with tumor, treatment, and socioeconomic and healthcare system characteristics. Transitions in care between institutions are responsible for the greatest increase in TTI. As TTI is more commonly used as a quality metric, physicians need to be aware of the causes for prolonged TTI, as we work to improve national delays in diagnosis and treatment initiation.

## 1. Introduction

On an annual basis, there are approximately 3,500 new patients diagnosed with primary bone sarcoma (PBS) in the United States [1]. While treatment almost always includes surgery, the addition of chemotherapy in the 1980s dramatically increased the survival [2–4]. Similar to other cancer types, it is recommended that treatment be initiated as early in the disease course as possible to reduce the risk of metastatic spread or growth [5–7].

Time to treatment initiation (TTI), defined as the time in days from histologic diagnosis of malignancy to initiation of definitive treatment, is being used as a quality control metric in an effort to improve patient outcomes in cancer referral centers. Prolonged TTI is reported to have a negative survival impact in several cancer types, including breast and head/neck cancer [8–10], lending evidence for expedited treatment strategies. To our knowledge, there have been no similar studies assessing the prognostic influence of TTI in PBS, yet it is a logically accepted mindset to expedite diagnosis and treatment with hopes of theoretically improving outcome. Thus, with a limited understanding surrounding the importance of time in the treatment of PBS, it is helpful to identify the risk factors associated with TTI in PBS.

Our primary aim was to quantify the current norms for TTI for PBS in the United States. Additionally, we aimed to identify patient, tumor, treatment, and healthcare-associated characteristics associated with prolonged TTI following a PBS diagnosis.

## 2. Methods

### 2.1. Database

Following the approval by our IRB, the National Cancer Database (NCDB) was reviewed from 2004 to 2013. Created in 1989 by the American College of Surgeons (ACS) and the Commission on Cancer (CoC), the NCDB captures 70% of all new United States cancer diagnoses, compiling data from over 1,500 cancer centers [11]. The requirements and methodology for reporting to the NCDB have been described [11–14].

### 2.2. Selection of Patients

Adult patients (≥18 years old) with PBS diagnosed between 2004 and 2013 were identified using topographical codes (C40.0-C40.3, C40.8-C41.4, C41.8, C41.9) designated by International Classification of Disease for Oncology, Third Edition (ICD-O-3). To be included, a patient also required an ICD-O-3 histology code consistent with a PBS. These codes identified a total of 15,083 patients with a PBS. Patients were excluded if they did not receive definitive treatment in the form of surgery, radiotherapy, systemic therapy, or other forms of treatment (*n*=1,720). Additional patients were excluded if TTI was >365 days (*n*=34) due to potential recording error or patient decision to voluntarily delay treatment.

Patient, tumor, treatment, and healthcare system factors were assessed to identify their relationship to TTI. Patient factors included demographics such as age, gender, race, Charlson/Deyo Score (0, 1, or ≥2), and annual income. Tumor and treatment factors included histologic subtype, primary site, size, grade, clinical stage, and initial definitive treatment modality. Healthcare system factors included treating facility type, insurance provider, distance from the patient's residence to the treating facility, and a transition in care institution. Facility types are divided into community cancer programs, comprehensive cancer centers, academic centers, integrated network cancer programs, and others types (i.e., Veteran's Affairs cancer programs). While both offering diagnostic and treatment services, community cancer programs diagnose 100–500 new cancer cases a year, and comprehensive and academic cancer programs diagnose >500 new cancer cases a year. “New cancer cases” are defined as all histologic diagnoses—not exclusively sarcoma. Initial definitive treatments include surgery, radiotherapy, systemic therapy, or a combination of treatments that start on the same day. Noncurative treatments were not considered initial definitive treatments—such treatments may include a palliative or hospice approach. Patients who received a diagnosis at one facility and were then transferred to another facility for definitive treatment initiation were considered to have a transition in care institution.

### 2.3. Statistical Analysis

In order to determine significant variances in TTI within a variable, the Kruskal–Wallis test was used for nonparametric univariate analysis. Multivariate analysis was performed using negative binomial regression models, containing all covariates to control for confounders. *P* values were considered significant if *P* ≤ 0.05. Incidence rate ratio (IRR) is defined in the following manner—for every 1-point increase in the independent variable, while holding all other variables in the model constant, the TTI rate (in days) would increase by a factor of that value. Statistical tests were conducted using Stata Version 14 (College Station, TX).

## 3. Results

**Figure 1:**
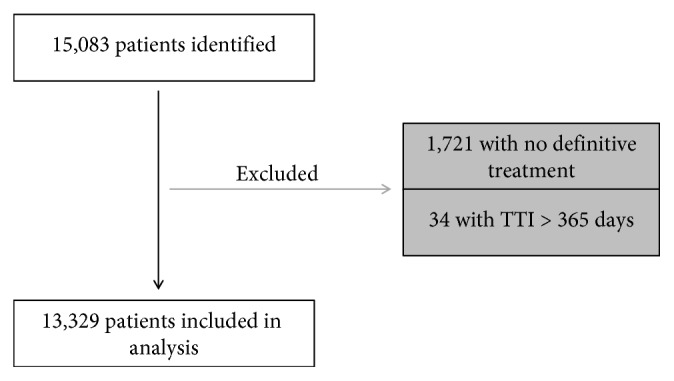
Inclusion and exclusion criteria (TTI, time to treatment initiation).

In total, 13,329 patients were included in the final analysis (Figure 1). Patients between 51 and 70 years of age (32%) were most commonly affected by PBS, followed by patients between 31 and 50 years (29%) and 18–30 years (24%). Over half of the population was male (56%), and the vast majority was either white (85%) or black (9%). The most common histologic types included chondrosarcoma (42%), osteosarcoma (30%), Ewing's sarcoma (10%), and chordoma (10%). The lower extremity (34%), pelvis (19%), and upper extremity (13%) were the most frequent sites of primary tumors. Academic centers (42%) and comprehensive cancer centers (15%) reported over half of the cases, while 40% of cases experienced a transition in care. Surgery (67%) and systemic treatments (26%) were the most frequent initial treatments.

**Figure 2:**
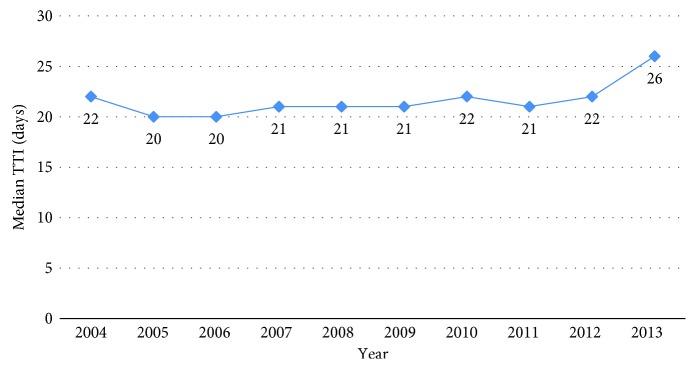
Median time to treatment initiation by year from 2004 to 2013. *P* value = 0.165 (TTI, time to treatment initiation).

**Figure 3:**
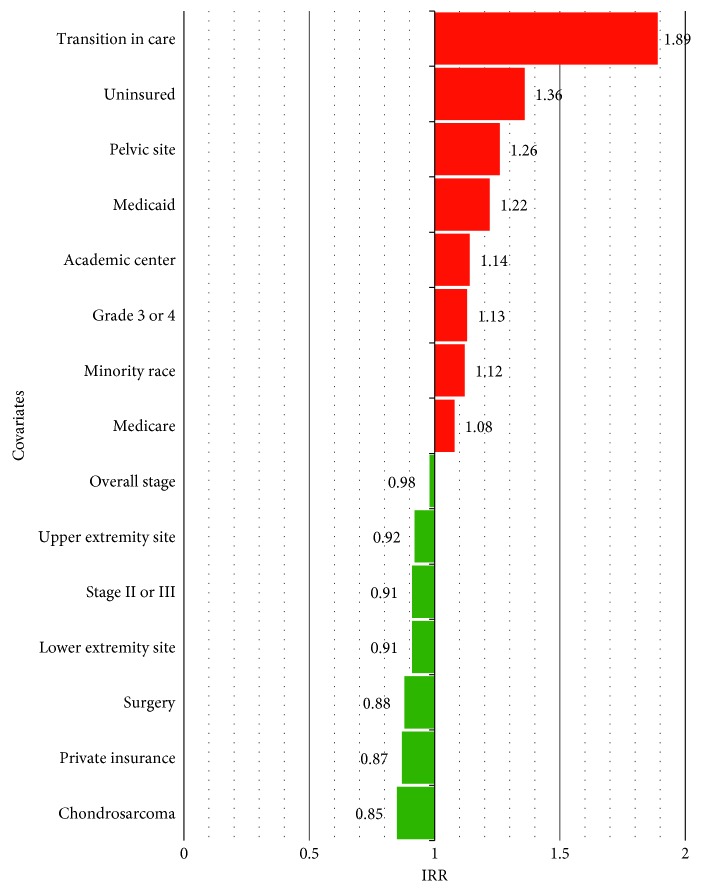
Comparison of relative association between covariates and time to treatment initiation. Only covariates with statistically significant higher (red) or lower (green) IRR are shown in this figure.

In the United States, the median TTI for all patients diagnosed with PBS was 22 days (interquartile range (IQR) 4–43 days), while the mean TTI was 32 days. From 2004 to 2013, median TTI remained relatively constant, with a slight increase in 2013 (*P*=0.165) (Figure 2). Univariate analysis revealed significant differences in TTI with regard to patient, tumor, and healthcare factors—including age, gender, race, histologic subtype, primary site, tumor size, grade, clinical stage, facility type, insurance, distance from facility, initial treatment, and transition in care (Table 1). A detailed report of the relative TTI IRR of each of these factors is displayed in Table 2.  Figure 3 compares the covariates associated with significantly increased TTI to those covariates associated with significantly shortened TTI.

The single patient factor that was significantly associated with TTI was race. Patients who were a minority race were found to have increased TTI compared to white patients (IRR = 1.12; 95% confidence interval (CI), 1.04–1.19; *P*=0.002). Age (IRR = 1.07; 95% CI, 0.94–1.14; *P*=0.051), female gender (IRR = 0.99; 95% CI, 0.94–1.04; *P*=0.612), and a Charlson/Deyo Comorbidity Score ≥1 (IRR = 0.98; 95% CI, 0.92–1.06; *P*=0.677) were not significant predictors of TTI.

Pelvic tumor location (IRR = 1.26; 95% CI, 1.19–1.35; *P* < 0.001) was associated with increased TTI compared to all other anatomical sites. Intermediate grade tumors (grade 2 (IRR = 1.24; 95% CI, 1.12–1.38; *P* < 0.001)) and higher grade tumors (grade 3/4 (IRR = 1.13; 95% CI, 1.03–1.23; *P*=0.006)) correlated with increased TTI compared to grade 1 tumors. Patients with chondrosarcoma had significantly decreased TTI compared to all other diagnoses (IRR = 0.85; 95% CI, 0.79–0.92; *P* < 0.001). Other tumor factors that significantly decreased TTI include upper extremity tumors (IRR = 0.92; 95% CI, 0.85–0.99; *P*=0.023) and lower extremity tumors (IRR = 0.91; 95% CI, 0.86–0.96; *P*=0.001) compared to all other primary tumor sites, and stage II/III (IRR = 0.91; 95% CI, 0.85–0.98; *P*=0.010) compared to all other stages. Larger tumor size (IRR = 1.00; 95% CI, 0.95–1.05; *P*=0.923), and an osteosarcoma diagnosis (IRR = 1.03; 95% CI, 0.97–1.09; *P*=0.316) did not significantly influence TTI.

Healthcare system factors were shown to independently affect TTI. Academic centers were associated with increased TTI compared to all other centers (IRR = 1.14; 95% CI, 1.08–1.21; *P* < 0.001) and especially when compared to only comprehensive cancer centers (IRR = 1.20; 95% CI, 1.11–1.30; *P* < 0.001). Compared to private insurance, uninsured (IRR = 1.36; 95% CI, 1.21–1.53; *P* < 0.001), Medicaid (IRR = 1.22; 95% CI, 1.12–1.33; *P* < 0.001), or Medicare (IRR = 1.08; 95% CI, 1.01–1.16; *P*=0.017) patients, all experienced significant TTI delays. A transition in care was the most significant predictor of increased TTI, when compared to patients without a transition in care (who received treatment at the same facility the diagnosis was established) (IRR = 1.89; 95% CI, 1.80–1.99; *P* < 0.001). Surgery as the initial definitive treatment was the only factor associated with significantly decreased TTI when compared to all other treatment types (IRR = 0.88; 95% CI, 0.84–0.94; *P* < 0.001). Living >20 miles from the facility did not significantly affect TTI compared to living ≤20 miles of the facility (IRR = 0.98; 95% CI, 0.93–1.03; *P*=0.520).

## 4. Discussion

TTI is gaining increased attention as a quality metric used by healthcare institutions for various cancer types, including lung, breast and head and neck cancer [8, 9, 15]. TTI initiatives are driven by patient demand, marketing, and the supposition that survival may be positively affected with expeditious treatment. Given the lack of reported TTI data in PBS, this study was intended to define the current national norms for TTI in adult patients with PBS in the United States, as well as to identify factors that may significantly increase or decrease TTI. This paper was not intended to link survival conclusions with TTI. These data show that, from 2004 to 2013, the median and mean TTI for PBS in the United States were 22 days and 32 days, respectively. As a comparison, TTI for head and neck cancer in the United States was 26 days between 1998 and 2011 [9], while TTI for colon [16] and lung [17] cancer in France between 2009 and 2010 was 22 and 34 days, respectively. In the current study, Figure 3 delineates those factors that were attributed to increased or decreased TTI in PBS. As has been previously noted with soft tissue sarcoma, the single largest factor associated with increased TTI is a transition in care [12].

A transition in care significantly increased median TTI by nearly three weeks (34 days vs 14 days). This longer time period may be explained by the process of scheduling an appointment, obtaining insurance approval, and transferring records, including imaging and pathology. In addition, Mankin et al. notably reported that changes in management were two to twelve times greater when a part of the diagnostic or treatment process was performed at a referring facility compared to entirely at a single treatment facility, which also could lead to longer treatment times [18, 19]. Further analysis of this dataset revealed an increasing proportion of PBS patients undergoing a transition of care in 2013 compared to 2004 (437/1189 = 36.7% in 2004 compared to 609/1383 = 44.0% in 2013; *P* < 0.001). This may signify a shift toward centralization of sarcoma cases to tertiary referral centers, which has been advocated [20–22].

While age, gender, comorbidities, income, and distance to the treating facility did not influence TTI, we found minority race and uninsured or government (Medicaid or Medicare) insurance status were all associated with increased TTI. Minority race and lower socioeconomic status are factors known to be associated with poorer access to care and worse survival in many types of cancer [23, 24]. Uninsured patients were less likely to receive treatment compared to their insured counterparts in melanoma [25] and hepatocellular carcinoma [26]. While insurance status has previously been shown to not be associated with the incidence of incompletely excised soft tissue sarcoma and appropriate referral patterns [27], recent studies have revealed black populations did not receive the same frequency of surgical resection or radiation therapy [28] and were more likely to receive amputations, when compared to white populations with soft tissue sarcoma [29]. Although the specific reason for these associations is unknown, they are likely multifactorial in nature.

Treatment at academic centers was associated with increased TTI by nearly a week compared to community cancer programs and comprehensive cancer centers. We hypothesize that this is not a reflection of trainee involvement, as referral practices and major treatment decisions are independent of the educational program. Increased TTI at academic centers is likely related to the “one-way street” pattern of referral from a community setting to a tertiary-care academic center—common in rare diseases. As indicated by this dataset, academic centers have seen an increasing proportion of PBS patients over the past decade (495/1189 = 41.6% in 2004 compared to 625/1383 = 45.2% in 2013; *P*=0.069), which may be due to the increased prevalence of multidisciplinary sarcoma teams in academic centers and associated education efforts to centralize referral to these teams.

Primary tumors of the pelvis were also associated with increased TTI. Tumors in the pelvis and sacrum can be an extensive surgical undertaking, often requiring long surgical time, multiple surgical teams, and complex surgical techniques [30, 31]. This additional coordination of care in pelvic sarcomas can undoubtedly lend itself to delays and the need for specialized resources—as evidenced by the fact that nearly half (46.7%, 1189/2543) of pelvic sarcomas were seen at academic institutions in this population. In contrast, extremity-based tumors were associated with decreased TTI, likely attributed to a more straightforward approach toward treatment.

Higher-grade tumors (grades 2–4) had an increased TTI compared to low-grade tumors (grade 1). One hypothesis is that more locally aggressive tumors may require more complex resection and extensive surgical planning, particularly if surrounding neurovascular structures are involved. Alternatively, low-grade tumors can often be biopsied and resected in the same setting (e.g., long bone low-grade chondrosarcoma [32]), lending to decreased TTI (often TTI = 0). With cartilage lesions, radiographic diagnosis may drive clinical suspicion to plan wide resection for higher-grade chondrosarcomas, with confirmatory frozen section at the time of surgery. This may offer an explanation as to why the diagnosis of chondrosarcoma and surgery as the index definitive treatment (includes excisional biopsies, TTI = 0 days) was associated with decreased TTI.

Lastly, it is interesting to note that though osteosarcoma and Ewing's sarcoma comprised 40% of this adult patient population, only 26% of patients had initial treatment with chemotherapy. Though the treatment specific data available through the NCDB did not offer any additional insight into this unique discrepancy, it is very thought provoking. One hypothesis for this finding may be as a result of the adult population (≥18 years old) in this study, with the theory that older osteosarcoma patients may be unable to tolerate chemotherapy to the same degree as younger patients, causing a larger proportion of patients to receive surgery as their initial treatment. Another factor that may contribute is that those with Paget's disease osteosarcoma are sometimes treated with up front resection prior to systemic therapy, given the aggressive nature of this variant.

This study has several limitations. First, data collection errors are inherent when using national registries, which can be attributed to incomplete and incorrect data found in medical charts. In addition, this NCDB search did not include patients <18 years old. This presents a selection bias away from certain subtypes of primary PBS, such as osteosarcoma and Ewing's sarcoma, as approximately 53% of osteosarcoma cases have been reported to occur in patients ≤24 years old [33]. Moreover, though the NCDB captures 70% of cancer cases, selection bias is possible in this database secondary to differences in institutions that report to the NCDB. In the previous literature, incidence for PBS has been reported to be 0.9 per 100,000 people/year [34]. Assuming this incidence with knowledge of the USA population from 2004 to 2013, the total estimated number of PBS between 2004 and 2013 was 27,450 [35]. This would suggest approximately 55% of all new PBS were reported to the NCDB in this time frame, making it less common to report than other more common malignancies, and harder to appreciate definitive conclusions from the current data. Despite these limitations, this study provides a large patient cohort to assess this novel topic in PBS care.

## 5. Conclusions

PBS has a median TTI of 22 days and a mean TTI of 32 days. In addition, socioeconomic, tumor, treatment, and healthcare system factors were found to significantly affect TTI. Potential causes of prolongation of TTI are multifactorial in nature. Using the information provided in this study, physicians can potentially identify their own institutional processes linked to treatment delays, with the goal to streamline treatment, improve patient care, and reduce patient anxiety. Future studies should assess the prognostic risk that delays in TTI pose on patient survival in PBS.

## Figures and Tables

**Table 1:** Comparison of patient, tumor, and healthcare factors on time to treatment initiation.

	Number of patients (%)	Median TTI, days (IQR)	*P* value
Total number of patients	13329	22 (4–43)	
Year of diagnosis			0.0002
2004	1189 (9)	22 (3, 43)	
2005	1339 (10)	20 (0, 42)	
2006	1258 (9)	20 (2, 41)	
2007	1307 (10)	21 (2, 45)	
2008	1290 (10)	21 (3.75, 42)	
2009	1393 (11)	21 (2, 43)	
2010	1370 (10)	22 (5, 46)	
2011	1349 (10)	21 (4, 43)	
2012	1451 (11)	22 (4, 43)	
2013	1383 (10)	26 (7, 48)	
Age (range)			0.0004
18–30 years	3220 (24)	19 (8, 35)	
31–50 years	3882 (29)	22 (0, 44)	
51–70 years	4248 (32)	23 (1, 47)	
71+ years	1979 (15)	24 (3, 48)	
Gender			0.0017
Male	7439 (56)	22 (6, 43)	
Female	5890 (44)	21 (0, 43)	
Race			0.0002
White	11277 (85)	21 (3, 42)	
Black	1228 (9)	25 (6, 49.75)	
Other/unknown	824 (6)	21 (3, 45)	
Charlson/Deyo score			0.5091
0	11433 (86)	22 (4, 43)	
1	1493 (11)	21 (1, 45)	
≥2	403 (3)	24 (0, 54)	
Income			0.4598
<$38,000	2216 (17)	22 (5, 46)	
$38,000–$47,999	3130 (24)	22 (4, 44)	
$48,000–$62,999	3504 (26)	21 (3, 42)	
$63,000+	4236 (32)	22 (2.25, 42)	
Unknown	243 (2)	21 (6, 40)	
Histology			0.0001
Osteosarcoma	3979 (30)	24 (10, 42)	
Chondrosarcoma	5608 (42)	19 (0, 43)	
Ewing's sarcoma	1313 (10)	19 (9, 32)	
Chordoma	1354 (10)	30 (1, 63)	
Other	1075 (8)	25 (7, 49)	
Primary tumor site			0.0001
Upper extremity	1743 (13)	20 (0, 40)	
Lower extremity	4482 (34)	20 (7, 37)	
Pelvis	2543 (19)	30 (13, 56)	
Other	4561 (34)	20 (0, 46)	
Tumor size			0.0001
≤8.0 cm	6397 (48)	21 (0, 44)	
>8.0 cm	6932 (52)	22 (7, 43)	
Grade			0.0001
1, well differentiated	2488 (19)	11.5 (0, 40)	
2, moderately differentiated	2246 (17)	26 (3, 49)	
3 or 4, poorly/undifferentiated	4300 (33)	23 (10, 41)	
Unknown	4295 (32)	22 (4, 45)	
Clinical staging			0.0001
Stage I	4419 (33)	24 (0, 49)	
Stage II	2518 (19)	25 (12, 42)	
Stage III	216 (2)	23.5 (10.25, 45)	
Stage IV	1288 (10)	21 (10, 38)	
Unknown	4888 (37)	19 (0, 42)	
Facility type			0.0001
Community cancer program	303 (2)	22 (0, 43)	
Comprehensive cancer center	2026 (15)	19 (0, 41)	
Academic center	5652 (42)	26 (5, 49)	
Integrated network cancer program	544 (4)	20 (0, 44)	
Other/unknown	4804 (36)	20 (6, 38)	
Insurance			0.0001
Uninsured	674 (5)	24 (5.75, 48)	
Private insurance	7488 (56)	20 (1, 41)	
Medicaid	1372 (10)	23 (8, 46)	
Medicare	2983 (22)	24 (3, 47)	
Other/unknown	812 (6)	28 (9, 52)	
Distance from facility			0.0001
<21 miles	6238 (47)	21 (1, 42)	
21–50 miles	2846 (21)	22 (5, 42)	
51–100 miles	1808 (14)	21 (5, 43)	
>100 miles	2212 (17)	25 (6, 48.75)	
Unknown	225 (2)	21 (6, 39.5)	
Initial treatment modality			0.0001
Surgery	8949 (67)	19 (0, 44)	
Radiation	765 (6)	37 (18, 68.5)	
Systemic	3500 (26)	24 (14, 38)	
Other	24 (0.2)	30.5 (2.5, 68.25)	
Multimodal	91 (0.7)	23 (6, 49)	
Transition in care			0.0001
Yes	5309 (40)	34 (17, 58)	
		Mean: 44.9 (43.7, 46.1)	
No	8020 (60)	14 (0, 33)	
		Mean: 23.4 (22.7, 24.1)	

Community Cancer Program volumes defined as 100–500 cancer cases annually. Integrated network cancer programs usually have a “unified cancer committee” and consist of “multiple facilities providing comprehensive services” [13]. Academic institutions are defined with the same quantitative volume definition as a comprehensive cancer center (>500 cancer cases annually) but also have a noted resident/medical education tract supported.

**Table 2:** Multivariate analysis.

	Incidence rate ratio on TTI (95% CI)	*P* value
Age (>30 years)	1.07 (1.00, 1.14)	0.051
Gender (female)	0.99 (0.94, 1.04)	0.612
Minority race	1.12 (1.04, 1.19)	0.002
Charlson/Deyo score ≥ 1	0.98 (0.92, 1.06)	0.677
Histology		
Osteosarcoma	1.03 (0.97, 1.09)	0.316
Chondrosarcoma	0.85 (0.79, 0.92)	<0.001
Primary tumor site		
Upper extremity	0.92 (0.85, 0.99)	0.023
Lower extremity	0.91 (0.86, 0.96)	0.001
Pelvis	1.26 (1.19, 1.35)	<0.001
Tumor size		
>8.0 cm	1.00 (0.95, 1.05)	0.923
Grade		
Overall grade	0.99 (0.97, 1.01)	0.265
Grade 2 vs grade 1	1.24 (1.12, 1.38)	<0.001
Grade 3 or 4 vs grade 1	1.13 (1.03, 1.23)	0.006
Clinical staging		
Overall stage	0.98 (0.98, 0.99)	<0.001
Stage II or III	0.91 (0.85, 0.98)	0.010
Facility type		
Academic center	1.14 (1.08, 1.21)	<0.001
Academic center vs comprehensive cancer center	1.20 (1.11, 1.30)	<0.001
Insurance		
Private insurance	0.87 (0.83, 0.92)	<0.001
Uninsured vs private insurance	1.36 (1.21, 1.53)	<0.001
Medicaid vs private insurance	1.22 (1.12, 1.33)	<0.001
Medicare vs private insurance	1.08 (1.01, 1.16)	0.017
Distance to facility ≥21 miles	0.98 (0.93, 1.03)	0.520
Initial treatment modality		
Surgery	0.88 (0.84, 0.94)	<0.001
Transition in care	1.89 (1.80, 1.99)	<0.001

## Data Availability

The data used to support the findings of this study are included within the article.
